# The Doctor Afloat

**Published:** 1894-07-21

**Authors:** 


### The Hospital

AN INSTITUTIONAL JOURNAL OF

The Medical Sciences and Hospital Administration,

WITH

AN EXTRA NURSING SUPPLEMENT.

VOL. XVI.—No. 408.] [JULY 21, 1894.

### THE DOCTOR AFLOAT.

WHILST it is possible to find individual doctors, and that in large numbers, who are entirely trusted and beloved by all who know them, it is still the case that the profession, as a whole, is not looked upon with a favour which is quite universal. But in the practical developments of modern times, not in civilised states only, but also in the outlying regions where civilisation is struggling with barbarism or science with the unscientific culture of bygone ages, the scientific doctor and health guide and teacher is found to be one of the most absolutely indispensable of all institutions. Wherever the Englishman has planted himself on foreign shores he has always taken his church with him, and in process of time his chapel. Now he takes, simultaneously, his doctor, and as soon as possible his hospital. It is clear then that in practice, as well as in theory, the modern health guide and consoler is destined to influence and, to some extent, to control all human life in its widest ramifications. It is desirable, therefore, that this medical permeating should be of the most competent and agreeable kind, and that those for whose benefit the permeation takes place should give it, not the shrug of a cold shoulder, but friendly encouragement and welcome.

A very considerable fraction of the time of all our middle and upper classes is spent at sea. Now the sea has many delights and many drawbacks. To some people the drawbacks much outweigh the delights. We do not hesitate to say, however, that if medical science could reign at sea for ten years, instead of being, like a notorious historical pretender to the English throne, a mere "scullion" in the ship's kitchen, many of the drawbacks would entirely disappear, and the delights would lose most of their alloy.

The ocean-going steamer may be looked upon as a town, the coasting vessel as a village or hamlet, and the yacht as one of the "stately homes" or "sweet country houses" of England afloat on the changeful deep. In each of these cases we have a mixed population, and all the necessities and dangers which attach to numbers and community of life. On shore the town, the village, and the "stately home" alike require government, order, cleanliness, sanitation; and the more intelligent the government and the more perfect the sanitation the greater is the happiness and well-being of each and every member of the community. If we say that this is almost exactly paralleled by life afloat, we are aware that we utter a truism of the tritest kind. But we utter the truism all the same, and emphasise it, because we know as a matter of fact and experience, that sanitation and medical guidance on board ship are often looked upon as extremely inconvenient impertinences, to be snubbed and kept, like the scullion in the kitchen, as much in the background as possible.

Of course it may be retorted upon us that if doctors and medical science are somewhat at a discount at sea, it is the fault of the doctors. It is said, with some show of justification, in the matter of sea-sickness, that the average doctor on shore can give directions before sailing which are found more serviceable in practice than the advice of the ship's doctor who spends his life on the deep. It is contended also that the ordinary ship's doctor is either so ignorant of ship-sanitation, or so cowardly in the carrying out of his duty, that he will see the most insanitary conditions, bearing danger and death everywhere, without uttering a single warning to passengers or crew, or a single protest to captain or owners. In short, it is said of ships' doctors, as it used to be said of parsons in the bad old times, that provided they find their stipends paid punctually they do not care so much as a brass farthing what becomes of the well-being of parish, or passengers, or crew.

If all these things be true, and some of them certainly are, it will give both relief and pleasure to many people to see an effort made to put an end to them. It would please us most to hear of some ships' doctors themselves starting up, like sanitary Martin Luthers, and resolving at all risks that the deadly insanitation of our mercantile and passenger fleets shall be "mended or ended." But the Luther of our floating towns and villages has yet to appear. In the meantime a kind of mild Melancthon, in the person of Dr. William Collingridge, has very opportunely put himself into the breach at the Port of London. Dr. Collingridge, as everybody knows, is medical officer to the port, and he made an excellent name for himself in connexion with his energetic and highly successful management of incoming vessels and their crews during the recent cholera scare. We shall never know how much of our immunity from cholera during the continental epidemic just passed we owe to Dr. Collingridge and other port medical officers of health. Emboldened perhaps by his success on that occasion, Dr. Collingridge has set himself to the task of condemning, and if possible of destroying, some as "hoary abuses" as ever disgraced the life story of a great sea-going people.

Dr. Collingridge has published a summary of his experience and advice in a short pamphlet, read in the first instance before the London Shipmasters' Society, under the title of "Practical Points on the Hygiene of Ships." It may be taken unreservedly for granted that the statements of facts put forward in the pamphlet are trustworthy, and free from exaggeration, for it is manifest that, since shipmasters are really responsible for the sanitary condition of ships, no statements too unfavourable to themselves would be allowed to pass unchallenged. In Dr. Collingridge's pamphlet, therefore, we may consider that we have a plain and unvarnished record of particulars in which, so far as the statements of facts are concerned, the most absolute confidence may be placed.

The "practical points," to which Dr. Collingridge gives the most prominence, are eminently those which require the seeing eye and firm sanitary hand of the trained doctor. What landsman, for example, would suspect that "bilge liquid," as we may call it, is often little better than the contents of a town sewer, and yet that, up to the present time, there is no uniform method of dealing with it in a systematic and effective way? In the hands of a doctor who knows his business and will do it, "bilge poison" can be as easily disposed of as the "slops" in a large hotel. In like manner, who would suppose that on the open sea sailors suffer most of all from those diseases which are engendered by impure air? An able-bodied seaman is allowed by law seventy-two cubic feet of space for his sleeping apartment; a patient in a modern hospital is allowed two thousand feet—more than twenty-seven times as much. In speaking on the subjects of "lavatory," "cleansing," "closets," and the like, Dr. Collingridge makes the curious remark that a sailor "certainly is not altogether beyond the pale of civilisation." What depths of degrading filthiness may be hidden beneath this strange but suggestive criticism, who shall say? Dr. Collingridge was addressing shipmasters. He was not, therefore, we may be sure, proclaiming their enormities upon the housetop. But to the man who is accustomed to the "inwardness" of modern methods of reproof these and similar mild-looking suggestions give peeps into whole worlds of misery and degradation which want but the touch of the Ithuriel's spear of modern medical illumination to stand revealed as a disgrace and a scandal to all who are in any way responsible for the condition of our British sailors and ships.

Those insanitary conditions which poison the bodies and destroy the lives of sailors cannot but be fatal in a corresponding degree to passengers and all on board. A ship is one structure; her inhabitants are one community. That air which is pestilential to one is pestilential to all. The sanitary deficiencies which make health impossible for the crew make it also impossible for the passengers. The doctor is needed: the cultured, scientific, courageous doctor. The doctor who will sacrifice his post and his emoluments rather than his conscience and his duty. This is the kind of doctor the public who go to sea should demand, and they should never be content until they see such a doctor installed with full authority in every sea-going ship.

